# Infertility in WNIN Obese Mutant Rats—Causes?

**DOI:** 10.5402/2011/863403

**Published:** 2011-12-15

**Authors:** Nemani Harishankar, Punjal Ravinder, K. Madhavan Nair, Nappanveettil Giridharan

**Affiliations:** ^1^National Centre for Laboratory Animal Sciences, National Institute of Nutrition, Indian Council of Medical Research (ICMR), Jami-Osmania PO, Andhra Pradesh, Hyderabad 500 007, India; ^2^Micronutrient Research Group, Biophysics Division, National Institute of Nutrition, Indian Council of Medical Research (ICMR), Hyderabad 500 007, India

## Abstract

We are maintaining two obese mutant rat strains (WNIN/Ob and WNIN/GR-Ob) in our animal facility since 1997. These rat colonies are perpetuated by crossing heterozygous littermates, since the obese phenotypes of both genders turned out be infertile. The present study revealed the reasons for this infertility. The male obese rats, though appeared normal in terms of sperm count, sperm motility and testis histology, however found wanting in terms of libido. This appeared to be due to low circulating testosterone levels seen in these animals, which should also account for low testis and accessory gland weights seen in them. The females exhibited delayed puberty, in terms of days taken for opening of vagina, irregular oestrus cycles and had small ovaries and short and stumpy uterine horns. The FSH peak observed in control lean animals during oestrus stage of the sexual cycle and also the E2 peak of normal oestrus cycle was conspicuously absent in these animals. They also showed elevated levels of progesterone throughout the sexual cycle. Thus the infertility seen in these mutants could be attributed to their abnormal gonadosteroid levels and the resulting anatomical and physiological defects.

## 1. Introduction

We are maintaining two obese mutant rat strains in our animal facility since 1997, derived from our Wistar (WNIN) stock. These are unique in certain aspects in comparison to similar rodent models of obesity established in the West [1–3]. The prominent differences are the presence of a unique “kinky tail” in homozygous obese (−/−) and heterozygous carriers (+/−) and the mode of its inheritance, that is, autosomal incomplete dominance [2]. However, like other obese rodent models reported so far, WNIN/Ob and WNIN/GR-Ob rats also show infertility [4]. So they are propagated by mating fertile heterozygous carrier (+/−) littermates, which produces three phenotypes: the homozygous lean (+/+), the heterozygous carrier (+/−), and the homozygous obese (−/−) in a 1:2:1 Mendelian ratio. The biochemical indices of obesity like insulin, triglyceride, cholesterol, and leptin were also found to be high in these strains [2, 3].

Altered reproductive function leading to infertility is normally seen in obese animals as well as humans, both in males and females. For example, in women, obesity is frequently associated with menstrual disturbances with a high risk of androgenic ovulatory dysfunction and polycystic ovary syndrome [5–9]. Obese men, on the other hand, exhibit low serum testosterone, and testosterone injections in such people were shown to restore fertility and also bring about weight reduction [10]. Concerning to animals, studies in obese male Zucker rats at various ages (4, 6, and 10 months) revealed inadequate sexual behavior [11], with low pituitary weight and low concentration of luteinizing hormone (LH) and follicle-stimulating hormone (FSH) [12, 13]. The animals also showed low testis weight, low levels of circulating testosterone, and low weights of levator ani (LA) muscle [14], which are androgen dependent.

In the present study, we looked for anatomical and physiological changes in the reproductive organs of these animals, to understand the reasons for their infertility. Gonad as well as the weights of other reproductive accessory glands in males females were noted and the levels of reproductive hormones like testosterone in males and LH, FSH, E2, progesterone, and prolactin in female rats of both the mutant strains were analyzed to see whether any of these parameters showed any drastic deviations compared to lean littermates.

The study was reviewed and approved by the Institutional Animal Ethical Committee (IAEC) and was conducted in accordance with the internationally accepted principles for laboratory animal use and care.

## 2. Methods

### 2.1. Animals, Procedures, and Parameters

A total of 48 weanling animals including of 6 male and 6 female from lean (+/+) and obese (−/−) phenotypes of both WNIN/Ob and WNIN/GR-Ob, were taken for the study. They were housed individually in standard polycarbonate cages with top grill having facilities for holding pelleted feed and drinking water in polycarbonate bottles with stainless steel sipper tubes (Techniplast, Italy). The animals were maintained at 22 ± 2°C, with 14–16 air changes per hour with a relative humidity of 50–60% and a 12-hour light/dark cycle. The animals were provided with sterile pelleted chow of standard composition established at our institute containing all the recommended macro- and micronutrients (56% carbohydrate, 18.5% protein, 8% fat, 12% fiber, and adequate levels of minerals and vitamins) needed for rats along with water, *ad libitum*.

Females from lean (+/+) and obese (−/−) phenotypes of both the strains were examined daily, for opening of vagina from the 21st day onwards. As soon as the vagina was opened, stages in estrus cycle were determined by preparing vaginal smears as described by standard procedures [15]. Smears were prepared in the morning to determine each stage of the estrus cycle, and blood was collected immediately through retroorbital route [16], from lean and at corresponding time points from obese rats as well. As soon as the animals reached 105 days of age, animals were sacrificed and ovaries and uterine horns were weighed. Males were fasted overnight, and blood was collected in the morning by retroorbital route as mentioned before. The blood was collected in plain tubes and centrifuged and the serum was prepared and samples were stored at −20°C, till their analysis.

### 2.2. Organ Weights and Sperm Count

At 105 days of age, animals were euthanized under CO_2_ inhalation. The male and female reproductive organs with accessory glands were separated from the viscera and cleaned from fat, blotted on a filter paper, and weighed (Sartorius analytical balance with 0.1 gm sensitivity). Additionally the length of the uterine horns was also measured by using a geometry scale. Levator ani (LA) muscle from lean and obese males was taken and weighed and expressed as wet weight [14]. The weight of the muscle was used as a bioassay for testosterone [17], since its weight correlated with androgen concentration. Extirpation of LA muscle was done by the modified technique of Rassaert et al. [18]. Sperms were collected from the cauda epididymis [19] and intact sperm cells were counted using a haemocytometer, and the counts were expressed as 10^6^ cells/mL.

### 2.3. Hormone Assay

Ovarian and testicular hormone levels were measured by radioimmunoassay (RIA) kits. LH and FSH kits were obtained from National Hormone and Pituitary Programme, National Institute of Health, Bethesda. Progesterone, estradiol, and testosterone were assayed using RIA kits (Diagnostic Products Corporation, Los Angeles, USA).

### 2.4. Statistical Analysis

Statistical analysis was carried out by using SSPS package version 5.1. Normally, Students *t*-test was used when comparing two groups and when more than two groups are involved; multiple ANOVA was carried out with multiple comparisons using Duncan's multiple range test analysis. All the values given were mean ± S.E. A *P* value of 0.05 was considered statistically significant.

## 3. Results

### 3.1. Pubertal Attainment and Reproductive Organ Weights

The days taken for vagina to open were significantly delayed in obese animals as compared to lean rats of WNIN/Ob group. Similar results were obtained with WNIN/GR-Ob group (Table 1). The reproductive accessory organ weights and other reproductive parameters of WNIN obese rats are given in Tables 1 and 2. The obese male showed significantly low epididymis and accessory gland weights compared to their lean littermates. There was also a significant difference in the weights of testis, compared to lean controls. However, the sperm counts were not different in both the mutants, compared to lean controls. The levator ani muscle weight was 2-3 times lower in obese rats (WNIN/Ob and WNIN/GR-Ob) in comparison to lean controls (Table 1). The epididymal fat weight was about two times higher in obese mutants of both groups, compared to their lean littermates. Ovary and uteri weights were reduced in obese animals, and the uteri were short and stumpy as well (Table 2).

WNIN/Ob and WNIN/GR-Ob obese mutant rats exhibited irregular pattern of estrous cycle extending to up to 8–12 days. The cycle was predominated by estrus and diestrus stages and in some animals anoestrus continued for a long time. The lean rats showed normal pattern of estrous cycle with typical stages (proestrus, estrus, metestrus, and diestrus) and the estrous cycle length was completed in 4 to 5 days (Table 2).

### 3.2. Reproductive Hormone Profile

The plasma testosterone levels of lean and obese rats are given in Table 1. The results are based on a single sample obtained at one time point of the day. The circulating testosterone levels of obese mutant rats were significantly lower than those of lean rats. Between the two obese mutants, WNIN/Ob rats had significantly higher levels of testosterone than WNIN/GR-Ob. The circulatory levels of progesterone and estrogen are given in Table 3. In lean rats, circulatory levels of progesterone and estrogen showed a characteristic pattern of rise and fall during the four stages of estrus cycle. In lean phenotypes, the progesterone levels showed a fall in estrus, followed by a rise in metestrus, while estrogen levels showed peak values during estrus with a fall in metestrus in both groups. No such pattern was seen in obese phenotypes of both groups, since these animals showed only two stages (estrus and diestrus). At these stages, while progesterone showed significantly (*P* < 0.001) high values compared to lean (except at diestrus stage in lean WNIN/GR-Ob), estrogen showed significantly low values. The lean control animals showed decreasing levels of prolactin at various stages of estrus cycle (Table 3). Prolactin levels were very high during estrous stage compared to their lean littermates in WNIN/Ob as well as in WNIN/GR-Ob mutants. Though during diestrus the levels came down, it was still high in obese rats. LH and FSH levels were measured in all the stages of estrus cycle in lean phenotypes, while it could be measured only in estrus and diestrus stages in obese phenotypes. No significant differences with respect to LH and FSH were seen between stages of cycle or between the lean and obese phenotypes of WNIN/Ob and WNIN/GR-Ob groups (Table 4).

## 4. Discussion

As mentioned in Section 1, our obese mutant rat colonies (WNIN/Ob and WNIN/GR-Ob) are maintained by mating heterozygous carrier (+/−) littermates. Though the male obese rats showed normal spermatogenesis as evidenced by cytology [2, 3], they were found to be lacking in libido as the animals were not interested in mounting, and even in the few cases which were successful, the vaginal plug without blood tinges was found mostly on the cage floor. This was similar to what was reported in Zucker fatty rats [20]. In WNIN obese females histopathological observations earlier had shown cystic ovaries and short and stumpy uteri [21]. In the present study we extended these observations further and also looked into the status of reproductive hormones.

With respect to obese males, the normal sperm count seen is in agreement with the normal spermatogenesis observed in cytological examination of testis [21]. However, it appears that the circulatory testosterone levels are just adequate for normal spermatogenesis and normal sperm function, but not sufficient enough to maintain normal weights of testis and accessory glands and provide mating stimulus to these animals. This is reflected in the lower weights of testis, seminal vesicle and LA muscle, and their failure to mount. The weights of seminal vesicle and LA muscle are often taken as a measure of testosterone concentration, and the latter in particular is suggested as a bioassay for testosterone [22]. Studies by Whitaker et al. [22] using Zucker rats, showed that, although LA muscle weights were significantly low seminal vesicle and testis weights did not seem to be affected. This could be due to the extent of testosterone reduction in these strains, which was about 30%, compared 50% reduction seen in the present mutants. However, to another study by Saiduddin et al. [23], in Zucker male rats, did report low testis weights without any mention of testosterone concentrations.

As was seen, greater reduction of circulating testosterone concentrations was observed in WNIN obese mutant rats compared to lean littermates. Though, this could be responsible for the reduction in the weights of accessory glands, as well LA muscle, testis function was not found to be affected by such a reduction. Diabetic BB (Bio-breeding) rat strain having 35% reduction in plasma testosterone showed testicular atrophy and this was found to be in proportion to the duration of diabetes in these rats [24]. A strong correlation between plasma testosterone and blood glucose was also seen in them [25]. As reported earlier, WNIN/GR-Ob rats are glucose intolerant but they do not show frank diabetes [3]. But the extent of testosterone reduction in these animals shows a pointer to a tendency for higher reproductive function impairment compared to WNIN/Ob mutants. It has been observed that WNIN/GR-Ob rats can be made “diabetic” by maintaining them on a purified carbohydrate diet [26], and, under such conditions, the extent of infertility could be as severe as that is reported in BB rats, a possibility that needs to be examined.

The reproductive problem of WNIN obese female mutants seems to be more severe, where the puberty is delayed considerably as seen by the days taken for opening of the vagina. The ovary and uteri were found to be atrophic and mostly nonfunctional, and the estrous cycle totally abolished. In fact, the delay in onset of puberty (76 days) and the length of estrous cycle (8.9 ± 1.02 in WNIN/Ob and in WNIN/GR-Ob 10.0 ± 2.01) are the maximum reported so far, compared to similar data reported in other obese rat models [24]. The cycle predominantly consisted mainly of two stages, estrus and diestrus, and many animals exhibited prolonged periods of estrus or diestrus and some anestrus, which persisted for a long time. Irregular estrous cycle and smaller uteri were reported in other obese rodents, like Zucker fa/fa rats [23, 27] and Ob/Ob and db/db mice [28]. However, this could not be attributed to any difference in circulating levels of 17-*β* estradiol [22]. But the finding of small uteri does indicate either low levels of estrogen or less response of uterus to circulating estrogen levels. The former was found to be true by the studies of [13] in which the weight of the uterus was found to be increased on treatment with a daily dose of 0.01 *μ*g to 0.1 *μ*g of estradiol. In Ob/Ob and db/db mice, the ova were found to be fertile, when they were transplanted in to lean Ob/Ob and db/db mice, and these animals produced viable litter as well [28].

In normal rats, estrogen and progesterone follow a typical pattern of rise and fall during the stages of estrus cycle, the estrogen especially reaching a peak just before estrus [29]. In the present obese mutants, no such pattern was seen and only two stages of estrous cycle could be identified, that is, estrus and diestrus. The progesterone levels were high and the estrogen levels were low in obese females at these stages compared to lean animals. LH and FSH levels in these animals were also found to be low at these stages, unlike Zucker rats, where the concentrations of these hormones were reported to be high [12]. Deficiencies in estrogen receptors *α* in the anteroventral periventricular nucleus and progestin receptors in the medial preoptic area in obese Zucker female rats were seen, and these were attributed to be responsible for the poor responsiveness to ovarian steroid hormones in these animals [30]. The prolactin levels in the present mutants were found to be high, and histopathological studies show that some of these animals do have polycystic ovaries [31]. Polycystic ovaries with high levels of prolactin tend to lead to infertility problems as mentioned earlier [31]. Thus, the abnormalities seen in the levels of these hormones may be the reason for the infertility in the present mutants. The response of these obese mutants to exogenous estradiol treatment and the viability of ova from the ovaries in corresponding lean littermates need to be investigated to get further insight into the problem.

Unlike male obese rats, the hormonal deficiencies in the females were to the same extent in both the mutants. In Ob/Ob mice, hypogonadism and infertility were shown to be related to decreased levels of FSH, LH, and testosterone compared to lean littermates [32]. The condition in these mice was attributed to the immature hypogonadal axis existing at the pubertal stage. This was supported by experiments in Ob/Ob mice, where they were found to be sensitive to exogenous testosterone, on the feedback inhibition of FSH and LH compared to the lean controls [33]. It will be worthwhile to measure the levels of LH and FSH in WNIN obese males, with and without testosterone administration to see whether defective feedback mechanism(s) at hypothalamus pituitary level exist in these animals too.

In conclusion, the data presented here clearly shows that, in both groups of mutants (WNIN/Ob and WNIN/GR-Ob), circulatory levels of androgens are affected and may be even the pituitary gonadotrophins. As a result of this hormonal deficiency, the weights of gonads and associated structures were effected and the affect is much severe in females than in males. It can be thus safely concluded that abnormal reproductive hormone levels seen in both the males and females are indeed responsible for the infertility seen in these mutants.

## 5. Addenda

Many of the references cited in this paper refer to the period of 1970–1980, the time when Ob/Ob and db/db mice and Zucker rats, the most widely used animal models worldwide, were being identified and characterized, anatomically and physiologically. Natural mutation is a rare phenomenon, and our model was identified and established during the 1990s [34]. But their gross characterization, with respect to the above parameters, was completed only during 2005, as we had to await the completion of 25 generations for the mutation to be stabilized and established. The nature of mutation in these animals is currently under investigation, and the available data indicate that the mutation is on chromosome no. 5, close to leptin receptor. Our earlier studies have shown that neither the leptin nor the leptin receptor was altered in these animals [35].

## Figures and Tables

**Table 1:** Reproductive indicators, estrous cycle, and pubertal attainment in WNIN female rats (*n* = 6).

Phenotypes	Body weight (g)	Ovary weight (g)	Uterine horn length (cm)	Estrous cycle in days	Pubertal attainment in days
WNIN/Ob +/+	346.53 ± 42.19	1.68 ± 0.06	3.48 ± 0.89	4.20 ± 0.80	38.30 ± 0.54
WNIN/Ob −/−	617.86 ± 86.5*	1.01 ± 0.22*	1.05 ± 0.24*	8.90 ± 5.02	75.60 ± 1.83*
WNIN/GR-Ob +/+	290.58 ± 7.93	1.10 ± 0.18	3.20 ± 0.14	4.24 ± 0.92	37.02 ± 0.24
WNIN/GR-Ob −/−	513.00 ± 20.32*	0.92 ± 0.14*	1.65 ± 0.16*	10.02 ± 2.01	77.01 ± 0.84*

Values are mean ± S.E. *P < 0.05 by Student's *t-*test.

**Table 2:** Reproductive accessory organ weights and plasma testosterone levels in WNIN obese male rats (*n* = 6).

Phenotypes	Testis wt. (g)	Sperm count cells/mL	Seminal vesicle. wt. (g)	Epididymal fat wt. (g)	LA muscle wt. (mg)	Testosterone ng/mL
WNIN/Ob Lean (+/+)	2.93 ± 0.13	3354 × 10^6^	1.56 ± 0.78	3.13 ± 0.94	161.00 ± 0.02	7.76 ± 2.08
WNIN/Ob Obese (−/−)	2.09 ± 0.06*	4004 × 10^6 !^	0.82 ± 0.009*	8.22 ± 1.09*	86.40 ± 0.004*	3.72* ± 1.78
WNIN/GR-Ob Lean (+/+)	3.01 ± 0.12	3968 × 10^6^	1.44 ± 0.08	3.53 ± 0.54	224.05 ± 0.82*	8.71 ± 11.33
WNIN/GR-Ob Obese (−/−)	2.55 ± 0.12*	3900 × 10^6 !^	0.70 ± 0.008*	7.62 ± 0.29*	78.02 ± 0.008	2.98* ± 2.1

Values are mean ± S.E. *P < 0.05 by Student's *t*-test. LA: muscle levator ani muscle. Wt.: weight.

**Table 3:** Plasma LH and FSH levels of WNIN obese female rats (*n* = 6).

Estrus stages	LH (ng/mL)				FSH (ng/mL)			
	WNIN/Ob		WNIN/GR-Ob		WNIN/Ob		WNIN/GR-Ob	
	Lean	Obese	Lean	Obese	Lean	Obese	Lean	Obese

Proestrus	0.94 ± 0.17	N.A	0.97 ± 0.59	N.A	4.20 ± 1.78	N.A	5.01 ± 1.02	N.A
Estrus	1.10 ± 0.52	0.86* ± 0.14	0.84 ± 0.3	1.22* ± 1.12	2.30 ± 0.72	4.00* ± 1.61	6.60 ± 1.69	3.30* ± 0.81
Metestrus	0.65 ± 0.38	N.A	0.58 ± 0.39	N.A	3.50 ± 1.52	N.A	7.20 ± 2.17	N.A
Diestrus	0.67 ± 0.25	0.59* ± 0.32	1.87 ± 0.8	0.88* ± 0.06	4.20 ± 1.32	3.60* ± 0.96	3.60 ± 1.22	4.60* ± 1.02

Values are mean ± S.E. *P < 0.05 by student “*t*” test. LH: luteinizing hormone; FSH: follicular stimulating hormone.

N.A: not applicable.

**Table 4:** Progesterone, estrogen, and prolactin levels in lean and obese female rats (*n* = 6).

Estrus stages	Progesterone (ng/mL)				Estrogen (pg/mL)				Prolactin (pg/mL)			
	WNIN/Ob		WNIN/GR-Ob		WNIN/Ob		WNIN/GR-Ob		WNIN/Ob		WNIN/GR-Ob	
	Lean	Obese	Lean	Obese	Lean	Obese	Lean	Obese	Lean	Obese	Lean	Obese

Proestrus	16.60 ± 6.16	N.A	27.80 ± 8.90	N.A	15.20 ± 2.40	N.A	13.86 ± 6.02	N.A	27.20 ± 2.90	N.A	12.20 ± 3.30	N.A

Estrus	8.70 ± 3.38	18.30* ± 6.80	46.00 ± 8.77	19.00* ± 3.12	38.80 ± 4.15	26.60* ± 6.48	3.12 ± 0.72	9.40* ± 1.76	16.70 ± 4.90	32.10* ± 4.35	13.60 ± 9.14	99.00* ± 6.33

Metestrus	12.20 ± 3.58	N.A	31.24 ± 10.80	N.A	21.40 ± 4.35	N.A	20.86 ± 2.43	N.A	9.41 ± 1.30	N.A	12.40 ± 7.67	N.A

Diestrus	10.00 ± 2.79	20.20* ± 6.89	41.25 ± 11.20	22.00* ± 3.60	21.60 ± 9.60	17.00* ± 2.85	29.50 ± 11.80	15.26* ± 2.80	5.70 ± 1.32	10.20* ± 2.46	20.60 ± 6.80	17.10* ± 2.23

Values are mean ± S.E. *P < 0.05 by student “*t*” test. N.A: not applicable.
